# Procyanidin B2 improves endothelial progenitor cell function and promotes wound healing in diabetic mice *via* activating Nrf2

**DOI:** 10.1111/jcmm.16111

**Published:** 2020-11-20

**Authors:** Jiawei Fan, Hairong Liu, Jinwu Wang, Jiang Zeng, Yi Tan, Yashu Wang, Xiaoping Yu, Wenlian Li, Peijian Wang, Zheng Yang, Xiaozhen Dai

**Affiliations:** ^1^ School of Basic Medicine Chengdu Medical College Chengdu China; ^2^ Experimental Research Center The First Affiliated Hospital of Chengdu Medical College Chengdu China; ^3^ Wendy Novak Diabetes Center Louisville KY USA; ^4^ Pediatric Research Institute Department of Pediatrics University of Louisville Louisville KY USA; ^5^ Department of Pharmacology and Toxicology University of Louisville Louisville KY USA; ^6^ Department of Clinical Laboratory Xinjiang Provincial Corps Hospital of Chinese People's Armed Police Urumqi China; ^7^ School of Public Health Chengdu Medical College Chengdu China; ^8^ School of Biosciences and Technology Chengdu Medical College Chengdu China; ^9^ Department of Cardiology The First Affiliated Hospital of Chengdu Medical College Chengdu China

**Keywords:** angiogenesis, endothelial progenitor cells, nuclear factor erythroid 2 (NF‐E2)‐related factor 2, procyanidin B2, wound healing

## Abstract

One of the major reasons for the delayed wound healing in diabetes is the dysfunction of endothelial progenitor cells (EPCs) induced by hyperglycaemia. Improvement of EPC function may be a potential strategy for accelerating wound healing in diabetes. Procyanidin B2 (PCB2) is one of the major components of procyanidins, which exhibits a variety of potent pharmacological activities. However, the effects of PCB2 on EPC function and diabetic wound repair remain elusive. We evaluated the protective effects of PCB2 in EPCs with high glucose (HG) treatment and in a diabetic wound healing model. EPCs derived from human umbilical cord blood were treated with HG. The results showed that PCB2 significantly preserved the angiogenic function, survival and migration abilities of EPCs with HG treatment, and attenuated HG‐induced oxidative stress of EPCs by scavenging excessive reactive oxygen species (ROS). A mechanistic study found the protective role of PCB2 is dependent on activating nuclear factor erythroid 2‐related factor 2 (Nrf2). PCB2 increased the expression of Nrf2 and its downstream antioxidant genes to attenuate the oxidative stress induced by HG in EPCs, which were abolished by knockdown of Nrf2 expression. An in vivo study showed that intraperitoneal administration of PCB2 promoted wound healing and angiogenesis in diabetic mice, which was accompanied by a significant reduction in ROS level and an increase in circulating EPC number. Taken together, our results indicate that PCB2 treatment accelerates wound healing and increases angiogenesis in diabetic mice, which may be mediated by improving the mobilization and function of EPCs.

## 1 INTRODUCTION

The incidence of diabetes mellitus (DM) is rapidly growing worldwide. Foot ulceration is one of the most common complications in DM patients,^1^ and emerging evidence shows that diabetic foot ulceration increases the mortality rate.^2^, ^3^ More than half of diabetic foot ulceration are infected and about 20% severe diabetic foot infections lead to amputation.^1^ Impaired wound healing is the critical factor responsible for diabetic foot ulceration.^4^ However, the mechanism by which diabetes impairs wound healing has not been well elucidated, and effective treatments are still lacking.

Wound healing is a complex and dynamic process; it may be divided into four phases, including hemostasis, inflammation, proliferation and wound remodelling.^5^ The wound healing process could be affected by various factors via interfering with one or more phases. The mechanism of impaired wound healing in DM is not well understood, but it is found that sustained inflammation and impaired angiogenesis are the major factors responsible for delayed wound healing in diabetes.^6^, ^7^ Angiogenesis is the beginning and the key step of the tissue growth phase. However, hyperglycaemia and hyperlipidemia in DM resulted in normal angiogenesis impairment.^8^ Endothelial progenitor cells (EPCs) are the precursors of endothelial cells and can differentiate into mature endothelial cells that directly contribute to postnatal neoangiogenesis and vascular endothelial repair.^9^, ^10^ However, the number of EPCs is decreased, and the function of EPCs is impaired in diabetes.^11^, ^12^, ^13^ Some studies have demonstrated that increased apoptosis and EPC dysfunction are implicated in delayed diabetic wound healing.^14^, ^15^ Promoting the survival abilities and function of EPCs may be a potential therapy for diabetic wound healing.

Procyanidins are polymers or oligomers of flavonoids that are usually present in a wide variety of natural products, including fruits and other plants.^16^ There is emerging evidence that procyanidins exhibit a variety of potent pharmacological activities, including anti‐oxidative stress, anti‐inflammation and glucose metabolism modulation.^17^, ^18^, ^19^ Procyanidin B2 (PCB2), a major component of procyanidins, has been reported to possess beneficial effects on diabetic complications in animal models.^20^, ^21^, ^22^ For example, grape seed PCB2 has been reported to have protective effects on diabetic nephropathy via inhibiting mesangial cells apoptosis^20^ and ameliorating podocyte injury.^22^ A previous study^23^ also showed that PCB2 protected against glycated low‐density lipoproteins induced vascular endothelial cells apoptosis by up‐regulation of L‐isoaspartyl methyltransferase. However, it is not clear whether PCB2 could promote EPC function contributing to diabetic wound healing.

In the present study, we investigated the effects of PCB2 on apoptosis, migration and tube formation abilities of EPCs under high glucose (HG)‐mimicked diabetic hyperglycaemic condition and dissected the underlying mechanisms. Furthermore, we evaluated the therapeutic potential of PCB2 in a streptozotocin (STZ)‐induced diabetic wound healing model.

## 2 MATERIALS AND METHODS

### 2.1 Isolation and culture of human EPCs from umbilical cord blood

Ethical approval for the collection of human umbilical cord blood from healthy newborns was granted by the Institutional Review Board of the First Affiliated Hospital of Chengdu Medical College. Fully informed consent was obtained from all the parents of the newborns prior to the collection of umbilical cord blood. Umbilical cord blood (20 ml) was mixed with 1 ml heparin (Sigma), and mononuclear cells (MNCs) were isolated by density gradient centrifugation using histopaque‐1077 (Sigma‐Aldrich) within 2 hours of sampling. EPCs were isolated from MNCs as described in a previous study.^24^ MNCs were suspended in endothelial growth factor‐supplement medium (EGM‐2 bullet kit, Lonza) with 10% foetal bovine serum (FBS) (Gibco) and seeded on 6‐well plates pre‐coated with 50 μg/mL fibronectin (Sigma‐Aldrich). Cells were maintained at 37°C with 5% CO_2_ in a humidified incubator. After 3 days of incubation, dead cells were washed away with phosphate‐buffered saline (PBS), and the new medium was added. Then, the medium was changed every 3 days. After 2 weeks of culture, cells were identified and used for the following experiments.

### 2.2 Identification of human EPCs

After 14 days of culture in endothelial‐specific medium,the non‐adherent MNCs were removed, and the adherent cells were characterized by Dil‐Ac‐LDL uptaking and ulex europaeus lectin‐1 binding assay according to our previous study.^25^ Briefly, the cells were washed with Dulbecco's phosphate‐buffered saline (DPBS) three times and incubated with 5 μg/mL Acetylated Dil lipoprotein from human plasma (Dil‐Ac‐LDL, Thermo Fisher Scientific) at 37°C for 4 hours. Then, the cells were incubated with 10 μg/mL fluorescein isothiocyanate‐labelled ulex europaeus lectin‐1 (FITC‐UEA‐1, Sigma‐Aldrich) for 1 hour at room temperature. After incubation, the cells were rinsed with DPBS for 3 times and were visualized by a fluorescence microscope (Olympus IX71, Olympus). Both FITC‐UEA‐1 and Dil‐Ac‐LDL positive cells were recognized as EPCs.

To further confirm the purity of these cells, flow cytometry assay was performed to analyse the expression of specific cell markers VEGFR2, CD133 and CD34. To block the nonspecific binding, cells were incubated with 5% bovine serum albumin (BSA, Sigma) for 30 minutes. After blocking, cells were stained with Alexa Flour®647 Mouse Anti‐Human VEGFR2 (1:50, BD Pharmingen^TM^), PE mouse anti‐human CD133 (1:50, BD Pharmingen^TM^) and FITC Mouse Anti‐Human CD34 (1:50; BD Pharmingen^TM^) antibodies at room temperature for 1 hour, respectively. The same fluoresce labelled isotype IgG served as a control to define the negative populations for each stain. Cells were analysed with BD Accuri C6 Flow cytometry (BD Biosciences), and data were analysed using FloJo software version 8.8.4 (TreeStar, Inc).

### 2.3 Cell apoptosis analysis

Endothelial progenitor cells seeded on 6‐well plates (1 x 10^5^ cells/well) were maintained under basal culture conditions with HG (33 mmol/L, Sigma Chemical Co.) or with a same dose of mannitol (Sigma Chemical Co.) as osmotic control for 24 hours in the absence or presence of different concentrations of PCB2 (Sigma‐Aldrich) (0.1 μmol/L, 0.5 μmol/L, 2.5 μmol/L). After treatment, EPCs were harvested by 0.25% EDTA‐free trypsin. The apoptotic percentage of EPCs was determined by APC‐conjugated Annexin V/propidine iodide (PI) Apoptosis Detection Kit (Biolegend, San Diego, CA) according to the manufacturer's instructions. The apoptotic EPCs were detected by flow cytometry. Early apoptotic cells were defined as AnnexinV^+^/ PI^–^.

### 2.4 Tube formation assay

Matrigel tube formation assay was used to evaluate the protective role of PCB2 on EPC angiogenic function under HG. Briefly, 96‐well plates were coated with growth factor‐reduced matrix gel (50 μL/well, BD Biosciences). EPCs (2 x 10^4^ cells/well) were suspended in 100 µL basal culture medium containing HG or the same dose of mannitol in the absence or presence of different concentrations of PCB2 (0.1 μmol/L, 0.5 μmol/L, 2.5 μmol/L) and seeded on the 96‐well pre‐coated with matrix gel. Tube structure formed after EPCs incubation at 37°C with 5% CO_2_ for 6 hours and microscopic images were acquired using an inverted microscopy (Nikon Eclipse E600, Nikon). The total length of tube structures in each well was calculated by Image J software.

### 2.5 Wound scratch assays

Wound scratch assay was used to evaluate the role of PCB2 in EPCs migration. EPCs were seeded on 6‐well plates (1 x 10^5^ cells/well) and cultured to 100% confluence. Sterile 200‐μL pipette tips were used to scratch a straight line in the cell layer to create a wound. Then, cells were washed with PBS and treated with basal culture medium containing HG or the same dose of mannitol for 12 hours in the absence or presence of different concentrations of PCB2 (0.1 μmol/L, 0.5 μmol/L, 2.5 μmol/L). Wound images were observed and photographed with light microscope. The wound closure rate was calculated by Image J software.

### 2.6 Quantitative determination of oxidative stress

Dihydroethidium (DHE; Molecular Probes) staining was used to detect reactive oxygen species (ROS) levels of frozen skin sections and EPCs according to the previous description.^11^ To evaluate the ROS level in wound skin section, frozen sections of the skin were harvested and stained with 5 μmol/L DHE in PBS for 30 minutes at room temperature. The ROS level in EPCs was determined as follows: firstly, EPCs were seeded on 24‐well plates and treated with mannitol or HG in the absence or presence PCB2 (2.5 μmol/L) for 48 hours. Then EPCs were rinsed twice with PBS and incubated with 5 μmol/L DHE for 30 minutes at room temperature. After twice washes with PBS, the fluorescent images of EPCs were captured by a fluorescence microscope (XI 71 Olympus), and the fluorescent intensity was analysed by Image J software.

### 2.7 Lentivirus infection

To knockdown Nrf2 expression in EPCs, EPCs were infected with the lentiviruses containing shRNA against Nrf2 (Nrf2‐shRNA‐EPC) or control nonsense shRNA (Ctrl‐shRNA‐EPC) constructed by Genechem. The transfection was performed according to the procedures described below: EPCs were infected with the lentiviruses at a multiplicity of infection (MOI) of 50 with 2.5 μg/mL polybrene (Genechem), and the medium was replaced with fresh growth medium 24 hours after infection. After transfection for 72 hours, the expression of Nrf2 was determined by qRT‐PCR and Western blot assay.

### 2.8 Cell counting kit‐8 assay

To confirm whether Nrf2‐shRNA lentivirus has cytotoxicity effects on EPCs, cell counting kit‐8 (CCK‐8) assay was performed to evaluate the viability of EPCs. After confirming the transfection efficiency of Nrf2‐shRNA, lentivirus‐infected EPCs (1 × 10^4^ cells/well) were seeded on 96‐well plate with 100 μL basal culture medium and incubated for 24 hours, then CCK‐8 (10 μL/well) solution was added to the culture medium, and the cells were further incubated for 2 hour. Finally, the absorbance value of medium was measured by microplate enzyme‐linked immunosorbent assay reader (Bio‐Tek Instruments).

### 2.9 STZ‐induced diabetes

Seven‐week‐old male C57/BL6 mice were purchased from Chengdu Dashuo Experimental Animal Company. All animal procedures were approved by the Animal Policy and Welfare Committee of Chengdu Medical College. All mice were maintained in a specific pathogen‐free facility at Chengdu Medical College with a 12‐h light/dark cycle and provided regular food and water for 1 week prior to any experimental procedures. Mice were randomly divided into two groups. In the diabetes group(n = 25), mice were intraperitoneal injected low‐dose STZ (50 mg/kg, Sigma‐Aldrich) in 0.1 mol/L sodium citrate buffer (pH 4.5) for 5 consecutive days to induce insulin deficiency and hyperglycaemia. While in the control group, mice were received an equivalent volume of citrate buffer. One week after the last injection of STZ, mice with a blood glucose level above 300 mg/dL were considered diabetic, whereas those with a level low 300 mg/dL received an additional STZ injection (50 mg/kg) for 1 day. Mice with a glucose level above 300 mg/dL for 4 weeks were considered diabetic and used in this study. Five mice died within 10 days after STZ injection in the diabetes group.

### 2.10 In vivo wound healing model and drug administration

The diabetic mice were randomly divided into either the diabetes control group (n = 10) or the PCB2 treatment group (n = 10). All control and diabetic mice were subjected to skin surgery according to the previous study.^26^ Mice were anaesthetized using 0.6% pentobarbital sodium (40 mg/kg), and the dorsal area was shaved. Two full‐thickness dermal wounds were made on both sides of the dorsal surface of each mouse with an 8 mm punch biopsy. PCB2 was dissolved in dimethyl sulphoxide (DMSO) at 100 mg/mL and then diluted with PBS. Mice in the PCB2 treatment group were received an intraperitoneal injection of PCB2 solution at a dose of 10 mg/kg daily from the day of surgery until the mice were euthanized, whereas mice in the remaining groups were administered an equivalent volume of PBS with the same dose of DMSO. At day 0, 1, 3, 5, 7, 10 after wound surgery, the wound surface area was recorded by a digital camera. The wound area was measured by ImageJ software to quantify the wound closure rate. At the end of the experiment, mice from each group were euthanized, peripheral blood was collected for EPCs analysis and lipid peroxides measurement, and tissue surrounding wounds were harvested for histological evaluation.

### 2.11 Malondialdehyde assay

The formation of malondialdehyde (MDA) is used as an indicator of lipid peroxidation. Thus, the concentration of MDA in peripheral blood was measured by MDA assay kit (Nanjing Jiancheng Inc) based on thiobarbituric acid reaction. The protocol was according to the manufacturer's instructions.

### 2.12 Quantification of circulating EPCs by flow cytometry

In adults, EPCs reside in the bone marrow (BM) niche that controls their survival, self‐renewal, differentiation, proliferation and mobilization. There are a lot of evidences showing that mobilization of EPCs from BM to peripheral blood is a key for neovascularization.^27^, ^28^ Accordingly, mobilization of EPCs from the BM to the peripheral circulation results in circulating EPCs increasing. Thus to detect whether PCB2 could promote EPC mobilization from bone marrow into peripheral blood, the number of EPCs in peripheral blood was detected by flow cytometry according to previous reports.^14^, ^25^, ^29^ At day 7 after surgery, blood samples were collected in 0.1 mol/L EDTA‐2Na‐coated tubes from the heart (n = 5 each group). PE‐conjugated Anti‐mouse CD34 and APC‐conjugated Anti‐mouse VEGFR2 antibodies (BD Pharmingen^TM^) were used to stain the whole blood at room temperature for 1 hour. Isotype control IgG (BD Pharmingen^TM^) was used to exclude false‐positive cells. After staining, the whole blood was lysed and fixed in FACS^TM^ lysing solution (BD Biosciences) for 5 minutes, then washed with PBS and centrifuged at 300 g for 5 minutes. The cells were suspended in 400µL PBS for flow cytometry analysis. VEGFR2 and CD34 double‐positive cells were defined as EPCs.

### 2.13 Haematoxylin and Eosin (H&E) staining

Skin tissues were fixed with 4% paraformaldehyde for 24 hours at room temperature, dehydrated with gradient ethanol, then embedded in paraffin, and cut into 5‐μm sections for staining to evaluate the wound closure area. The sections were stained with haematoxylin (0.2%) and eosin (1%) (H&E) to assay the pathological changes of wound skins.

### 2.14 Tissue immunofluorescence staining

Skin tissues were embedded in OCT and cut into 10‐μm sections for staining to evaluate the capillary density. After washing with PBS, tissue sections were blocked using 5% BSA for 30 minutes at room temperature, and then incubated with Alexa Fluor® 594 conjugated isolectin GS‐IB4 antibody (Thermo Scientific, Waltham, MA) for 1 hour, and nuclei were recognized by 4',6‐ diamidino‐2‐phenylindole (DAPI) staining. Fluorescent images were taken using a fluorescence microscope. The capillaries were counted in randomly selected fields. The capillary density is presented as the capillary number per field.

### 2.15 Western blot assay

Western blot was performed as described in a previous study.^11^ Wound skin tissues and harvested cells were lysed in ice‐cold RIPA lysis buffer (Santa Cruz Biotechnology). The protein concentration was determined using a Bradford protein assay kit (Bio‐Rad). The total proteins were separated on 10% sodium dodecyl sulphate polyacrylamide gel electrophoresis (SDS‐PAGE) and transferred to PVDF membranes (Millipore). The membranes were blocked in tris‐buffered saline with 5% non‐fat milk and 0.5% BSA for 1 hour, and then incubated with primary antibody overnight at 4°C, followed by incubation with the secondary antibodies for 1 hour at room temperature after standard washing procedures. The primary antibodies against different target proteins were purchased from commercial companies listed below: β‐actin (1:3000, Bioss Biotechnology), Nrf2 (1:1000, Abcam), NAD(P)H dehydrogenase quinone 1 (NQO‐1, 1:1000, Abcam), catalase (1:1000, Cell Signaling Technology); 3‐nitrotyrosine (3‐NT, 1:2000, Millipore), and 4‐hydroxynonenal (4‐HNE, 1:2000, Alpha Diagnostic International). All horseradish peroxidase (HRP)‐conjugated secondary antibodies were purchased from Bioss Biotechnology. Blots were visualized with Chemiluminescent HRP substrate (Millipore) and quantified with Quantity 5.2 software System (Bio‐Rad).

### 2.16 Quantitative real‐time PCR (qRT‐PCR)

Total RNA was extracted using total RNA extraction kit (Solarbio) and reversed transcribed using an iScript cDNA synthesis kit (Bio‐Rad) following the manufacturer's protocol. The primers of Nrf2, catalase, NQO‐1 and GAPDH were designed and synthesized from Shanghai Shenggong Inc. qRT‐PCR was performed with SYBR Green Supermix (Bio‐Rad), which contains 5 μL Supermix, 2 μL H_2_O, 2 μL cDNA and 1 μL primer. The comparative cycle time (Ct) method was used to determine fold differences between samples, and the expression of target genes was normalized to GAPDH as an endogenous reference (2^−△△Ct^).

### 2.17 Statistical analysis

All data are presented as mean ± SD. Statistical analysis was performed using Graphpad Prism version 8.0 (GraphPad Software Inc) with one‐way ANOVA, followed by post hoc multiple comparisons with the Scheffe' test. Statistical significance was considered as *P* < .05.

## 3 RESULTS

### 3.1 Characterization of human umbilical cord blood EPCs

Mononuclear cells isolated from human umbilical cord blood were plated on fibronectin‐coated culture dishes and cultured in EGM‐2 medium. After 14 days of culture, most cells were double‐positive of Dil‐Ac‐LDL and FITC‐UEA‐1, which were defined as EPCs (Figure S1A). Furthermore, flow cytometric analysis showed that these cells expressed high levels of the EPC‐specific markers VEGFR2, CD133 and CD34 (Figure S1B).

### 3.2 PCB2 improves the angiogenic function, survival and migration of EPCs treated with HG

**Figure 1:**
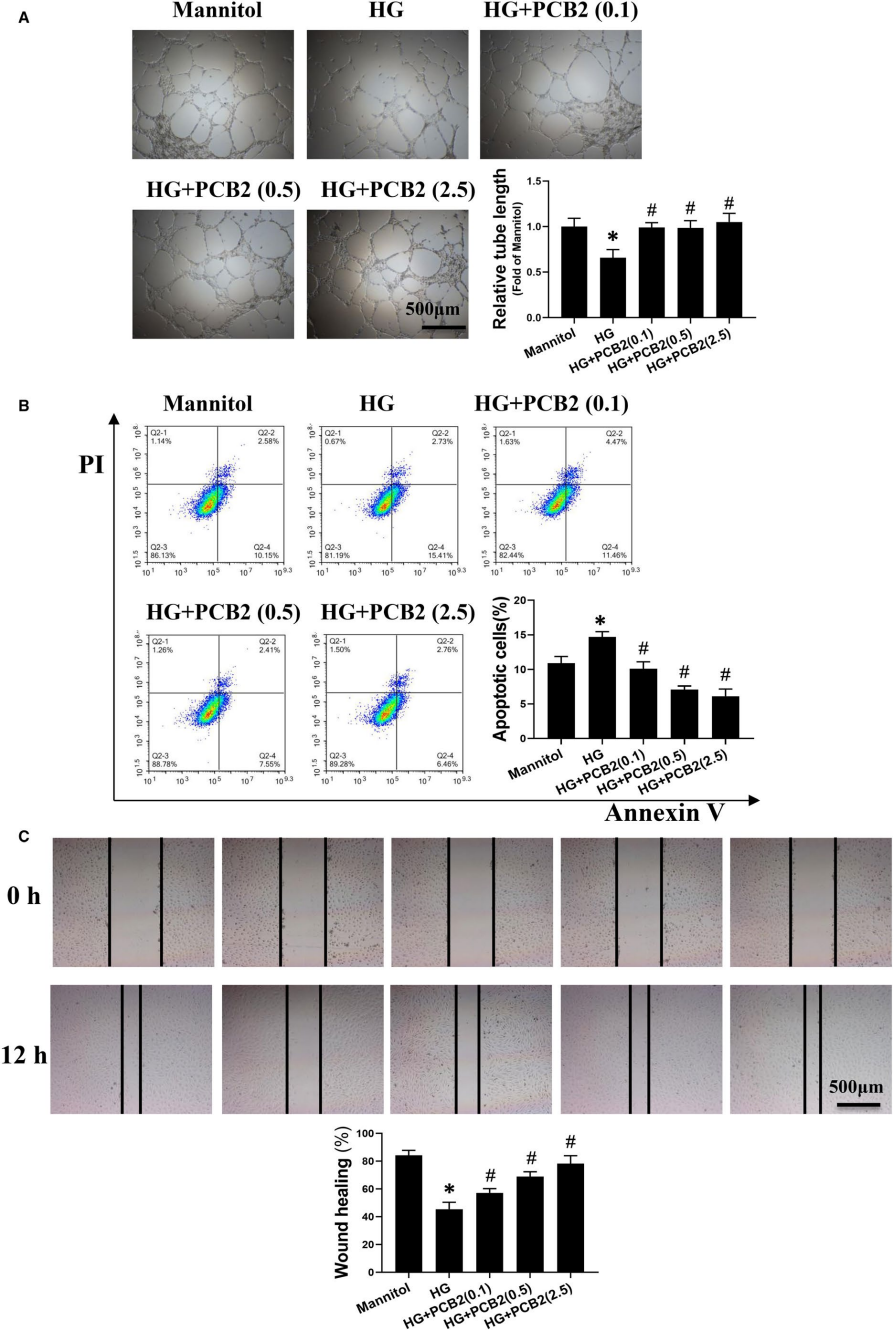
PCB2 improves the angiogenic function, survival and migration of EPCs treated with high glucose (HG). EPCs were exposed to HG (33 mmol/L) or with a same dose of mannitol as osmotic control in the absence or presence of different doses of PCB2. (A) The effects of PCB2 on the angiogenic function of EPCs were determined by tube formation assay after exposure to HG for 6 h. The tube length was measured by Image J and normalized to the mannitol control group. (B)The apoptosis of EPCs treated with HG for 24 h was detected by flow cytometry with Annexin V/propidium iodide (PI) staining. Early apoptotic cells were defined as AnnexinV^+^/PI^–^. (C) A scratch wound‐healing assay was performed to evaluate the effects of PCB2 on EPC migration. The migration distance was assessed at 0 and 12 h following cell‐scratching. The wound closure rate was calculated by Image J. Three independent experiments were performed for each study. Data shown in graphs represent the means ± SD. * *P* < .05, vs mannitol control group; ^#^ *P* < .05, vs HG treatment group

The results of matrigel tube formation assay showed that the tube formation abilities of EPCs were significantly impaired under HG condition, while PCB2 treatment significantly protected the angiogenic function of EPC against HG impairment (Figure 1A). Furthermore, the survival abilities of EPCs were determined by Annexin V/PI staining. Similarly, HG significantly induced EPC apoptosis, and PCB2 treatment prevented HG‐induced apoptosis of EPCs in a dose‐dependent manner (Figure 1B). Finally, the migration abilities of EPCs were evaluated by wound scratch assay. The results showed that HG significantly inhibited EPC migration, while PCB2 promoted EPCs migration and accelerated wound healing rate in a dose‐dependent manner (Figure 1C). These results indicate that the optimum dose of PCB2 protecting EPCs against HG is 2.5 μmol/L, which will be used in the following experiments.

### 3.3 PCB2 alleviates oxidative stress of EPCs treated with HG

**Figure 2:**
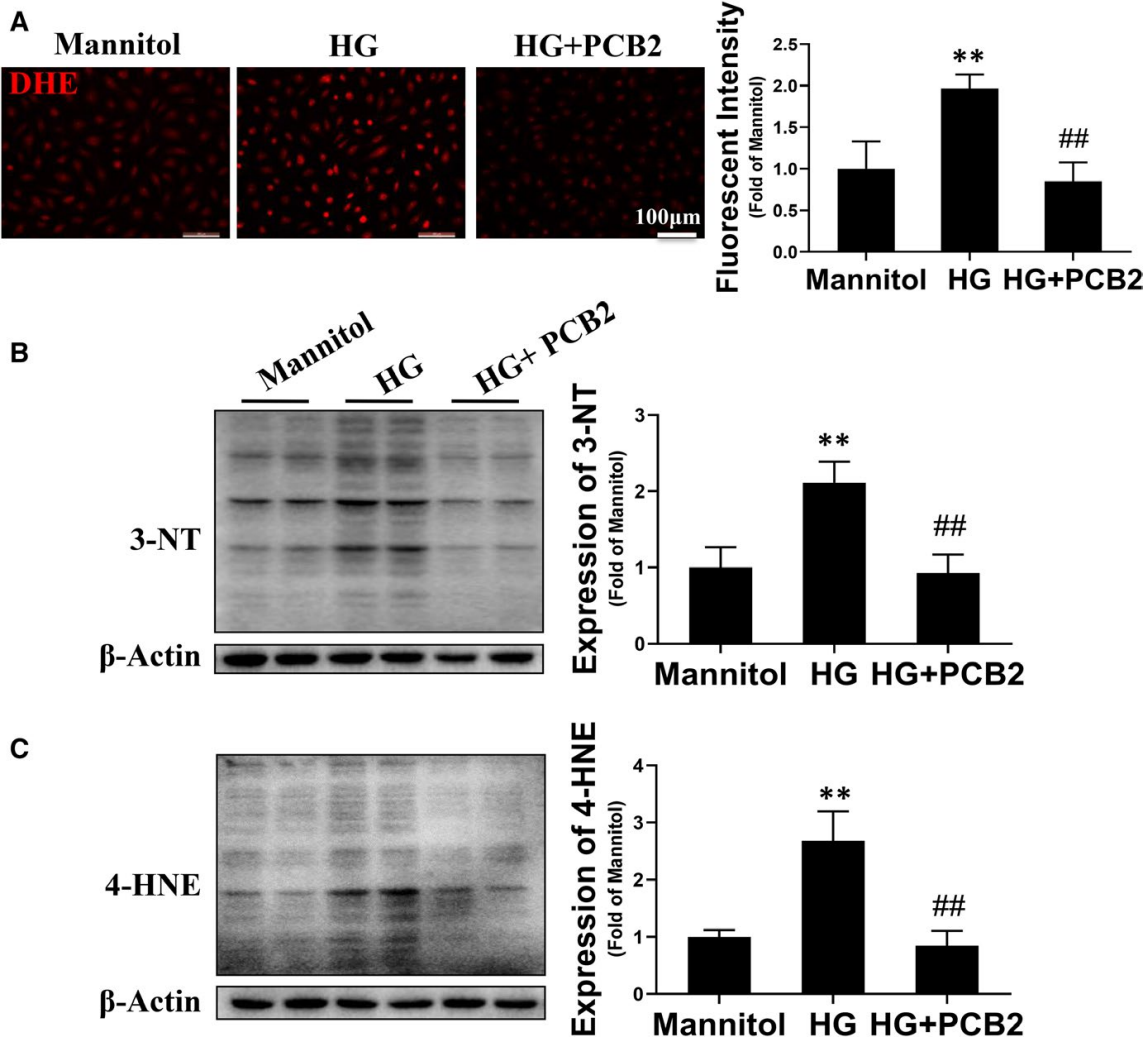
PCB2 attenuates HG‐induced oxidative stress in EPCs. EPCs were exposed to HG (33 mmol/L) or with a same dose of mannitol as osmotic control in the absence or presence of PCB2 (2.5 μmol/L). (A) The reactive oxygen species (ROS) level was detected with fluorescent indicator DHE staining; the fluorescent images were captured and the fluorescent intensity was measured by image J. The expression of oxidative damage markers 3‐NT (B) and 4‐HNE (C) was detected by Western blot. Three independent experiments were performed for each study. Data shown in graphs represent the means ± SD. * *P* < .05, ** *P* < .01 vs mannitol control group; ^#^ *P* < .05, ^##^ *P* < .01 vs HG treatment group

In our previous studies, we found that oxidative stress may be a major factor responsible for the dysfunction of diabetic EPCs.^11^, ^25^ PCB2 has been present elegant anti‐oxidative stress abilities.^30^, ^31^ However, it is not clear whether PCB2 could alleviate the oxidative stress of EPCs under diabetic conditions. In this study, DHE staining was used to detect the intracellular ROS level of EPCs. The results showed that the ROS level was significantly increased in EPCs treated with HG, which was remarkably attenuated by PCB2 treatment (Figure 2A). In addition, the oxidative damage markers (3‐NT and 4‐HNE) were determined by Western blot assay. The similar results were showed that PCB2 treatment almost completely inhibited the expression of 3‐NT and 4‐HNE induced by HG treatment in EPCs (Figure 2B,C). These results indicate that PCB2 can protect EPCs against HG‐induced oxidative stress.

### 3.4 PCB2 attenuates HG‐induced oxidative stress via activating Nrf2 signalling in EPCs

**Figure 3:**
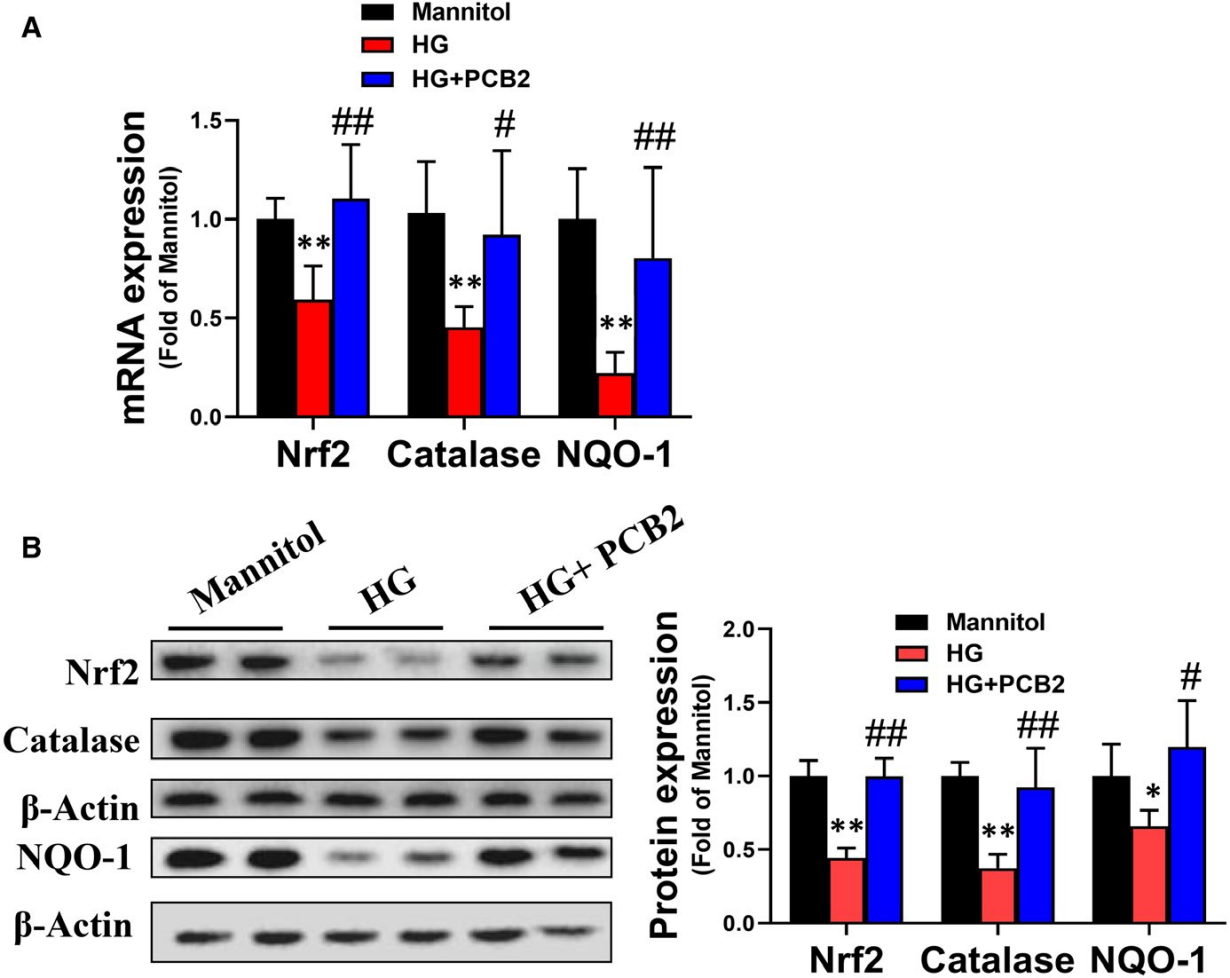
PCB2 attenuates HG‐induced oxidative stress in EPCs via activating Nrf2. EPCs were exposed to HG (33 mmol/L) or with a same dose of mannitol as osmotic control in the absence or presence of PCB2 (2.5 μmol/L). **(**A)The mRNA level of Nrf2 and its downstream genes (catalase and NQO‐1) were determined by real‐time PCR. (B) The protein levels of Nrf2 and its downstream genes (catalase/NQO‐1) were detected by Western blot. Three independent experiments were performed for each study. Data shown in graphs represent the means ± SD. * *P* < .05, ** *P* < .01 vs mannitol control group; ^#^ *P* < .05, ^##^ *P* < .01 vs HG treatment group

Nrf2 is a stress‐responsive transcription factor that involves the cellular response to oxidative stress.^32^, ^33^ To determine whether PCB2 attenuates HG‐induced oxidative stress via activating Nrf2 in EPCs, the expression of Nrf2 and its downstream genes were detected. The results demonstrated that HG treatment significantly decreased the expression of Nrf2 and its downstream genes catalase and NQO‐1 at both mRNA and protein levels (Figure 3). However, PCB2 treatment significantly prevented HG‐induced decreases of Nrf2 and its downstream gene expression (Figure 3).

### 3.5 Knockdown of Nrf2 impairs the protective effects of PCB2 on EPC function

**Figure 4:**
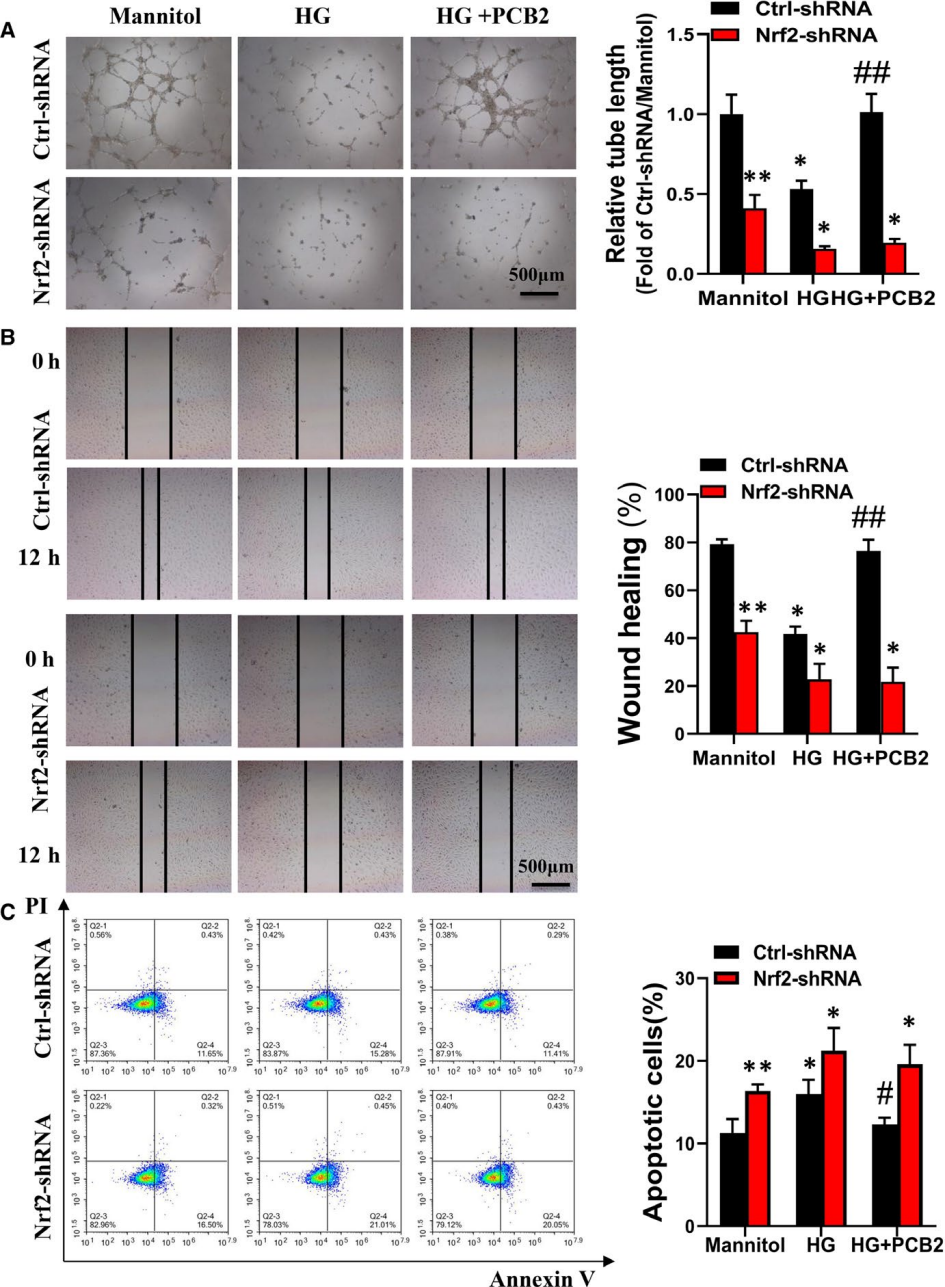
Knockdown of Nrf2 attenuates the protective effects of PCB2 on EPCs treated with HG. EPCs were transfected with lentivirus vector encoding Nrf2 shRNA (Nrf2‐shRNA‐EPCs) or control shRNA (Ctrl‐shRNA‐EPCs). Following shRNA transfection, the protective effects of PCB2 on EPCs angiogenesis, survival and migration abilities were determined as described in Figure 1. (A) Angiogenic function was determined by tube formation assay. (B) Apoptosis was analysed by flow cytometry. (C) The migration abilities of EPCs were performed by scratch wound‐healing assay. Three independent experiments were performed for each study. Data shown in graphs represent the means ± SD. * *P* < .05, ** *P* < .01 vs respective control in Ctrl‐shRNA‐EPCs or Nrf2‐shRNA‐EPCs; ^#^ *P* < .05, ^##^ *P* < .01 vs respective HG in Ctrl‐shRNA‐EPCs or Nrf2‐shRNA‐EPCs

**Figure 5:**
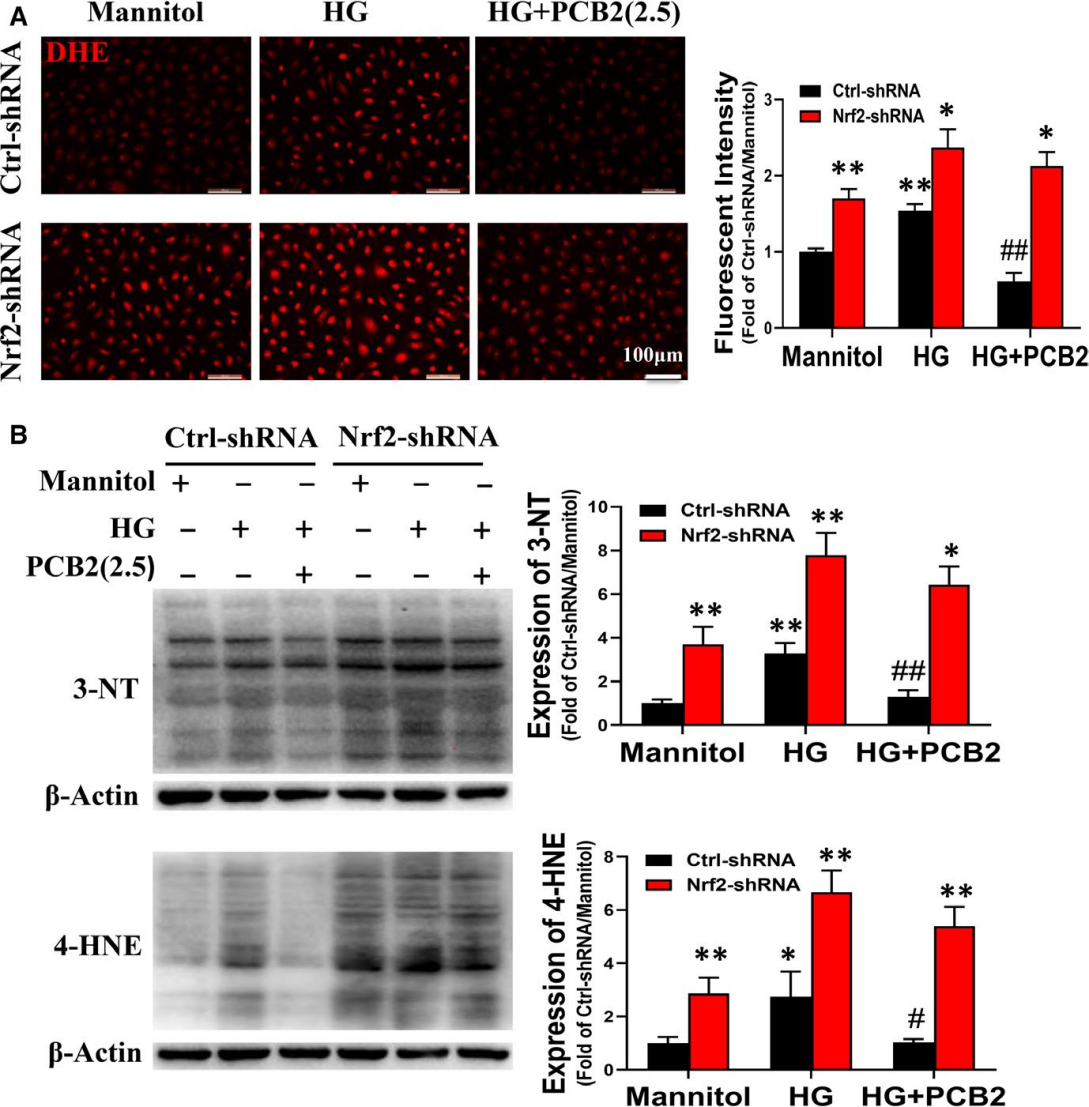
Knockdown of Nrf2 abolishes the anti‐oxidative effects of PCB2 on EPCs treated with HG. The effects of Nrf2 knockdown on the anti‐oxidative stress of PCB2 in EPCs were determined as described in Figure 2. (A**)** ROS level was determined by DHE staining. The expression of 3‐NT (B) and 4‐HNE (C) was detected by Western blot. Three independent experiments were performed for each study. Data shown in graphs represent the means ± SD. * *P* < .05, ** *P* < .01 vs respective control in Ctrl‐shRNA‐EPCs or Nrf2‐shRNA‐EPCs; ^#^ *P* < .05, ^##^ *P* < .01 vs respective HG in Ctrl‐shRNA‐EPCs or Nrf2‐shRNA‐EPCs

To confirm whether PCB2 improves EPC function under HG via activating the Nrf2 signal pathway, we performed a loss‐of‐function study by knockdown of Nrf2 gene expression with Nrf2‐shRNA lentivirus. To confirm the knockdown efficiency of Nrf2‐shRNA on EPCs, the expression of Nrf2 and its downstream genes was determined by Western blot and qRT‐PCR. The results demonstrated that Nrf2‐shRNA significantly decreased the expression of Nrf2 and its downstream genes, while the control nonsense shRNA (Ctrl‐shRNA) did not alter Nrf2 and its downstream genes expression (Figure S2A,B). As shown in Figure 4, Nrf2‐shRNA impaired the tube formation, survival and migration abilities of EPCs in mannitol group, which means Nrf2 knockdown may impair the viability of EPC. To confirm whether Nrf2‐shRNA has cytotoxicity effects on EPCs, we detected the viability of EPCs by CCK‐8 assay. The results showed that Nrf2‐shRNA transfection slightly decreased the viability of EPCs (Figure S2C), which means Nrf2 knockdown has a little cytotoxicity effect on EPCs. Furthermore, we found Nrf2‐shRNA eliminates the protective effects of PCB2 on tube formation, apoptosis and migration abilities of EPCs with HG treatment (Figure 4). Likewise, the superoxide production and the expression of oxidative damage markers (3‐NT and 4‐HNE) are also higher in the Nrf2‐shRNA group than that in the Ctrl‐shRNA group (Figure 5). Meanwhile, the expression of Nrf2 and downstream genes (catalase, NQO‐1) in Ctrl‐shRNA‐EPCs or Nrf2‐shRNA‐EPCs under high glucose with or without PCB2 was detected, the results showed that knockdown of Nrf2 significantly inhibited Nrf2 activation induced by PCB2 (Figure S3). Therefore, these results indicate that the protective effect of PCB2 against HG‐induced damage in EPCs is dependent on Nrf2 activation.

### 3.6 PCB2 enhances angiogenesis in dermal wounds and accelerates wound healing in diabetic mice

**Figure 6:**
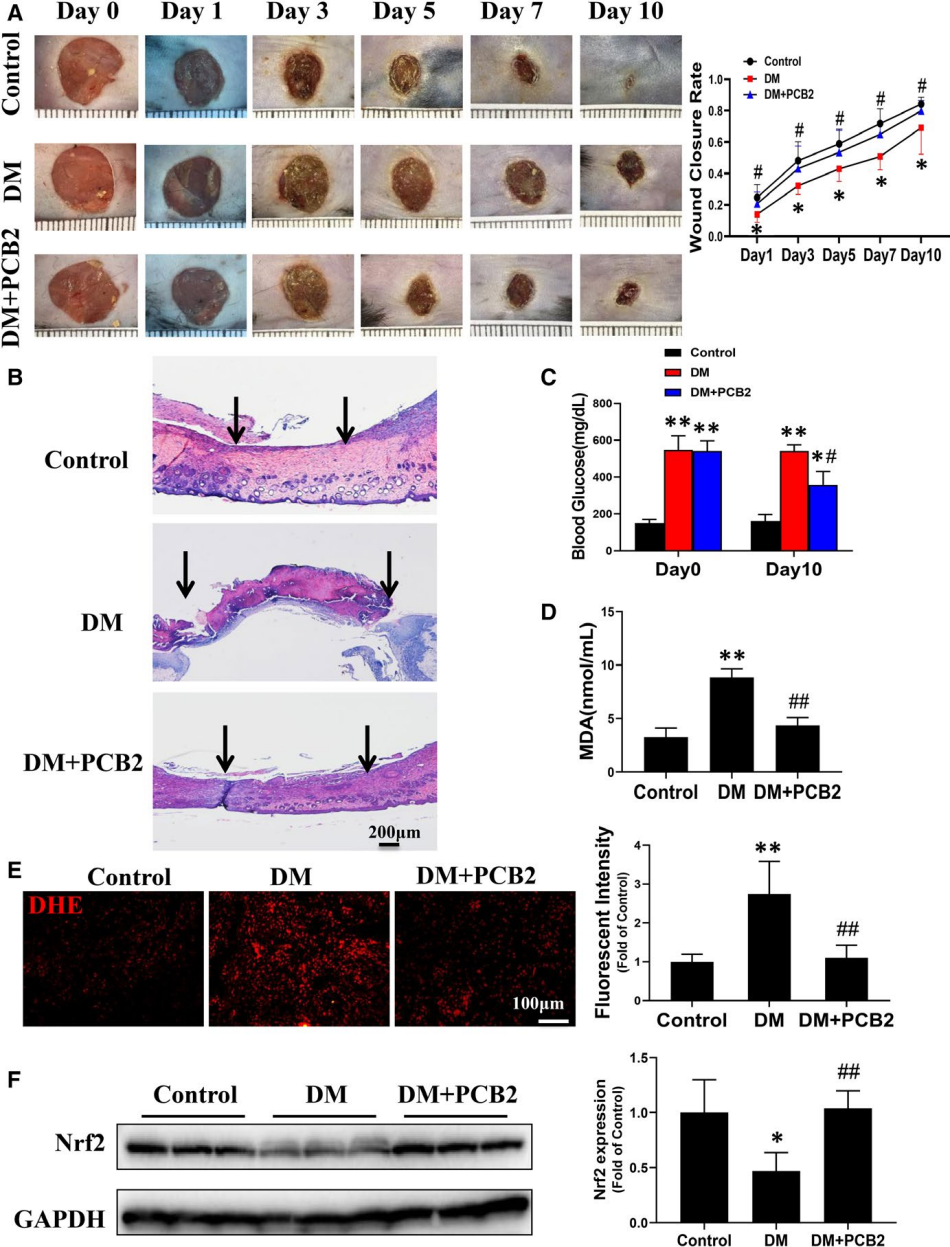
PCB2 accelerates wound healing and attenuates oxidative stress in diabetic mice with dermal wounds. (A) Representative images of excisional wounds of mice in different groups at the indicated time points. The wound area was measured by image J to calculate the wound closure rate. (B) Haematoxylin‐eosin staining of dermal wound tissues on day 10 post‐injury. The arrows indicated the edges of the wounds, where the epithelium was not yet formed. (C) The blood glucose levels in mice on day 0 and day 10 after injury. (D) The malondialdehyde (MDA) contents in mice serum on day 10 post‐injury. (E) Frozen sections of excisional wounds were used to evaluate superoxide production by DHE staining on day 7 post‐injury. The fluorescent intensity was quantified by Image J. n = 5 mice per group. (F) The protein expression of Nrf2 in dermal wound tissues was detected by Western blot. Data shown in graphs represent the means ± SD. * *P* < .05, ** *P* < .01 vs Control, ^#^ *P* < .05, ^##^ *P* < .01 vs DM. DM, diabetes mellitus

Considering the role of EPCs in the wound healing process is via promoting angiogenesis and vasculogenesis. To clarify whether PCB2 could improve EPCs function to promote diabetic wound healing, we examined the beneficial effects of PCB2 on wound healing in STZ‐induced diabetic mice. A standard in vivo wound‐healing assay was performed, and time‐dependent wound closure was determined. As shown in Figure 6A, diabetes significantly delays the wound closure rate compared to non‐diabetic control mice, while PCB2 treatment significantly accelerates the wound closure rate of diabetic mice. Histological analysis of the cross‐section by H&E staining showed the wound gap, the distance of between the advancing edges of epithelial layers at day 10 post‐surgery. The results demonstrated that PCB2 treatment decreased the distance of wound gap and promoted re‐epithelization in diabetic mice group (Figure 6B). Likewise, PCB2 treatment reduced serum glucose level in diabetic mice (Figure 6C). In addition, we evaluated the anti‐oxidative efforts of PCB2 on diabetic mice by detecting the MDA levels in peripheral blood and DHE staining in dermal wound tissues. The results showed that PCB2 treatment significantly decreased the MDA levels of peripheral blood and superoxide production of dermal wound tissues in diabetic group (Figure 6D,E), which means PCB2 could decreased oxidative stress in diabetic mice. We further detected the expression of Nrf2 in dermal wound tissues. The result demonstrated that the expression of Nrf2 in diabetic group was significantly decreased, while PCB2 treatment could preserve the expression of Nrf2 in diabetic mice (Figure 6 F). These results may indicate that PCB2 eliminates oxidative stress via activating Nrf2 signal pathway.

**Figure 7:**
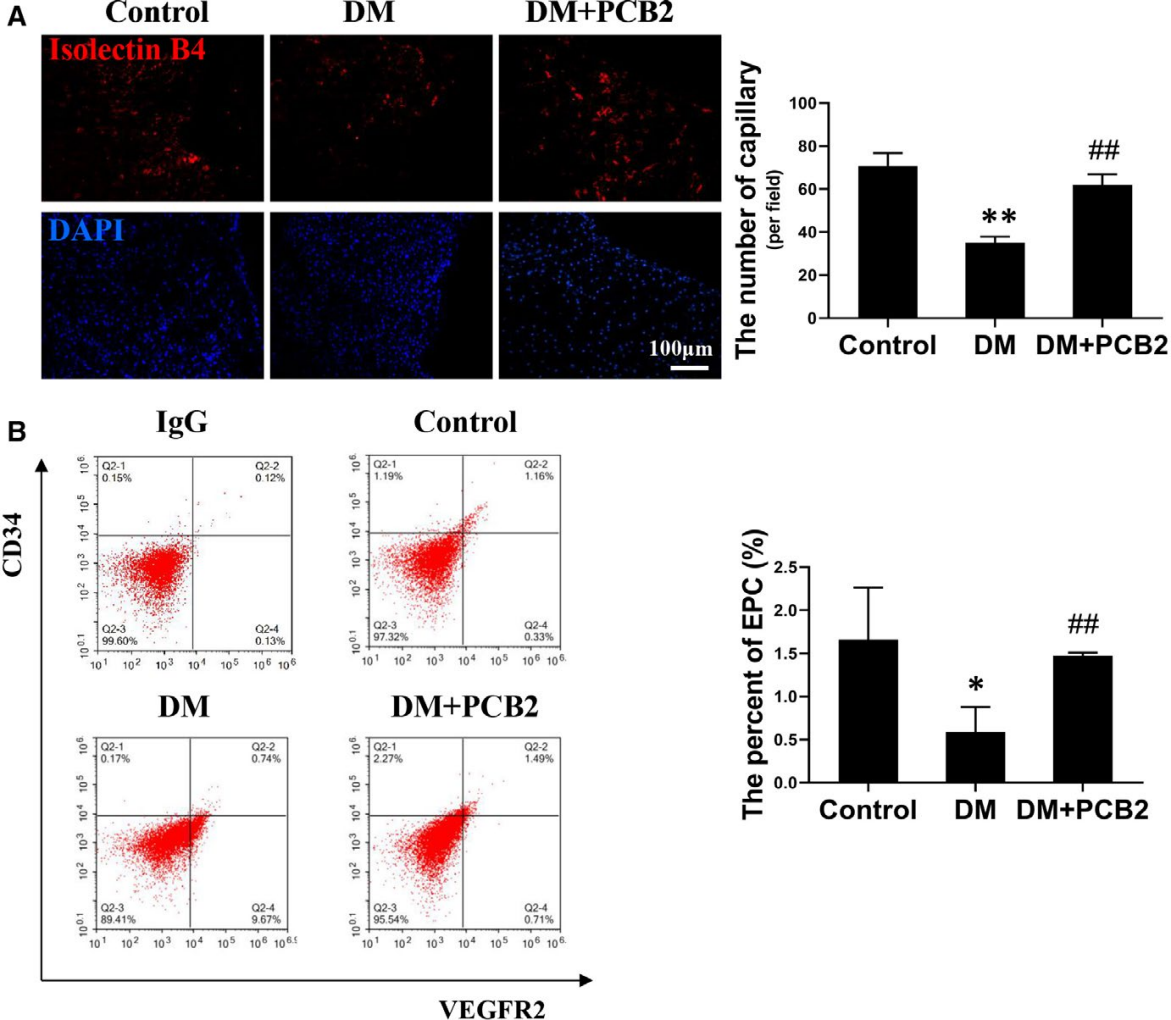
PCB2 enhances angiogenesis and increases EPC mobilization in diabetic mice with dermal wounds. (A) Transverse sections of excisional wounds were stained by isolectin GS‐IB4 to evaluate angiogenesis on day 10 after surgery. n = 5 mice per group. Capillary density was quantified as isolectin‐positive capillaries per field. (B) On day 7 after surgery, peripheral blood was collected to evaluate the number of EPCs (CD34^+^/VEGFR2^+^) in circulation by flow cytometry assay. n = 5 mice per group. Data shown in graphs represent the means ± SD. * *P* < .05, ** *P* < .01 vs Control, ^#^ *P* < .05, ^##^ *P* < .01 vs DM. DM, diabetes mellitus

To test whether PCB2 treatment promotes angiogenesis in diabetic dermal wound sites, the capillary density is determined by isolectin staining. The results showed that diabetes significantly attenuated capillary formation after dermal wound surgery, while PCB2 treatment significantly increased the capillary density (Figure 7A). Considering the role of EPCs in angiogenesis, peripheral blood is collected to determine the number of EPCs in circulation at day 7 after surgery. The flow cytometry assay shows that the number of circulating EPCs (CD34^+^/VEGFR2^+^) significantly decreases in the diabetes group, while PCB2 treatment significantly preserves the circulating EPC number compared to diabetic mice (Figure 7B). The results indicate that PCB2 can preserve diabetes‐attenuated EPC mobilization from bone marrow to peripheral blood after a dermal injury.

## 4 DISCUSSION

In the present study, we demonstrate the benefits of PCB2 in the angiogenic function of EPCs and wound healing in diabetic mice. The results show that PCB2 protects HG‐induced impairment in angiogenic function, survival and migration of EPCs by eliminating HG‐induced oxidative stress. Furthermore, the mechanism data demonstrate that the protective effect of PCB2 on EPCs under diabetic conditions is dependent on Nrf2 activation. Finally, we report that PCB2 accelerates wound healing and angiogenesis in STZ‐induced diabetic mice is associated with preserving the number of EPCs in circulation.

Despite advancements in wound care, chronic diabetic wound healing remains a great challenge in clinical therapy. Diabetic patients often display wound healing impairment, which leads to the development of foot ulcers and eventual amputation.^4^ One of the phenotypes of chronic diabetic wounds is associated with a lack of angiogenesis.^34^ EPCs can participate in angiogenesis via differentiating into mature endothelial cells or secreting pro‐angiogenic factors. Unfortunately, the number of EPCs is significantly reduced, and the function of EPCs is impaired in DM, which may result in poor angiogenesis outcomes in diabetic patients. Therefore, EPCs‐based therapy may be a promising approach for diabetic wound healing via EPCs transplantation or modulating the endogenous EPCs under diabetic conditions.^34^, ^35^ Despite Sukmawati et al demonstrated that oxidative stress was not the major initiating factor of vasculogenic dysfunction of BM‐EPC in early diabetes.^36^ There are several researches have reported that diabetes‐induced oxidative stress is one of the major contributors to dysfunction of EPCs and impaired endothelial regeneration.^11^, ^37^, ^38^ The controversy about the role of oxidative stress in diabetes‐induced EPCs dysfunction may be due to the different cell source and diabetic duration. Usually, the ROS level in circulating EPCs from long‐time diabetes is increased and causes EPCs dysfunction.^39^Thus, anti‐oxidative agents may be a potential strategy for improving EPC function.

Procyanidin B2 is a natural dietary phytochemical with multiple activities. Previous studies have demonstrated that PCB2 has anti‐inflammation, anti‐tumour and cardiovascular protective properties partially due to its anti‐oxidative effects.^21^, ^23^, ^40^, ^41^, ^42^ Yang et al found that PCB2 attenuated LPS‐induced production of reactive oxygen species (ROS) in human vascular endothelial cells.^21^ In this study, we found that PCB2 significantly prevented HG‐induced EPC apoptosis, angiogenic dysfunction and migration impairment (Figure 1), which coincided with previous findings that procyanidin promoted the proliferation and tube formation abilities of EPCs under HG conditions.^43^ Furthermore, we found that PCB2 reduced the ROS accumulation and decreased the expression of oxidative damage markers (3‐NT and 4‐HNE) in EPCs induced by HG (Figure 2). These results indicate that PCB2 ameliorates the survival and migration abilities and angiogenic function of EPCs under HG conditions as a scavenger in oxidative stress.

Nrf2 is a stress‐responsive transcription factor and involved in the cellular response to multiple stressors.^33^ Nrf2 binds to regulatory antioxidant response elements and regulates the expression of antioxidant genes to scavenge ROS.^44^ Some studies have shown that grape seed proanthocyanidin extract alleviates oxidative damage in mouse testis and liver by activating Nrf2 signalling.^45^, ^46^ Another research also found that PCB2 ameliorated carrageenan‐induced chronic nonbacterial prostatitis via activating Nrf2 signal pathway.^47^ In our study, the results showed that PCB2 markedly increased Nrf2 and its downstream antioxidant target genes expression in EPCs with HG treatment (Figure 3). These results indicated that PCB2 improved EPC antioxidant capacity by activating Nrf2 transcriptional function. Our previous study reported that the activation of Nrf2 in EPCs from diabetic mice was decreased, elevating CXCR7 protected EPCs against diabetes‐induced injury *via* activating the Nrf2 signal pathway.^11^ Furthermore, we found the protective role of PCB2 was dependent on Nrf2 activation; knockdown of Nrf2 almost completely abolished the benefits of PCB2 on EPCs survival, tube formation and migration (Figure 4). In addition, the effects of PCB2 on ROS attenuation were nearly abolished by Nrf2‐shRNA (Figure 5). Therefore, these findings indicate that PCB2 prevents HG‐induced impairment of EPC function and survival is predominantly mediated by activating the Nrf2 signalling pathway to scavenge ROS. However, the mechanism of how PCB2 activates Nrf2 in EPCs under HG condition needs further examination.

In the diabetic wound healing model, we found that the wound closure rate was slower in diabetic mice compared to control mice. However, PCB2 treatment accelerated the healing process and decreased the oxidative stress in wound skin tissues (Figure 6). In addition, we found the expression of Nrf2 in diabetic wound skin was decreased, while PCB2 could preserve the expression of Nrf2, which may indicate PCB2 attenuates oxidative stress of wound skin via activating Nrf2. Furthermore, we evaluated the angiogenesis of wound skin by calculating the density of capillaries. The results showed that there were more capillaries in PCB2‐treated diabetic mice group than that of diabetic mice group (Figure 7A). These results suggested that PCB2 could improve diabetic wound healing and promoted neovascularization after dermal injury. While the mechanism by which PCB2 improves diabetic wound healing is not clear. Several studies have reported that effective treatment for diabetic wound healing is attributable to EPC mobilization to the wound site for neovascularization.^15^, ^48^ Furthermore, the results showed that PCB2 increased the number of EPCs in circulation, demonstrating that PCB2 could promote EPCs mobilization (Figure 7B). However, how PCB2 promotes EPCs mobilization needs to be further investigated.

In conclusion, PCB2 improves the survival, migration and angiogenic function of EPCs under HG conditions, which are predominantly due to the activation of the Nrf2 signalling pathway. PCB2 treatment accelerates wound healing and neovascularization in STZ‐induced diabetic mice by increasing the number of EPCs in circulation.

## CONFLICT OF INTERESTS

The authors confirm that there are no conflicts of interest.

## AUTHOR CONTRIBUTION

**Jiawei Fan:** Methodology (lead). **Hairong Liu:** Methodology (lead). **Jinwu Wang:** Methodology (equal). **Jiang Zeng:** Methodology (equal). **YI TAN:** Writing‐original draft (equal). **Yashu Wang:** Data curation (equal). **Xiaoping Yu:** Writing‐review & editing (equal). **Wenlian Li:** Data curation (equal). **Peijian Wang:** Data curation (equal); Writing‐review & editing (equal). **Zheng Yang:** Project administration (equal); Writing‐original draft (equal). **Xiaozhen Dai:** Funding acquisition (lead); Project administration (lead); Writing‐original draft (lead).

## Supporting information

Figure S1

Figure S2

Figure S3

## Data Availability

The data supporting the findings of this study are available from the corresponding author [X. D.] upon reasonable request.
