# Supplementary figures and images for: Procyanidin B2 improves endothelial progenitor cell function and promotes wound healing in diabetic mice via activating Nrf2
Source: J Cell Mol Med. 2020 Nov 20;25(2):652–65. doi: 10.1111/jcmm.16111 (PMC7812287; doi:10.1111/jcmm.16111)

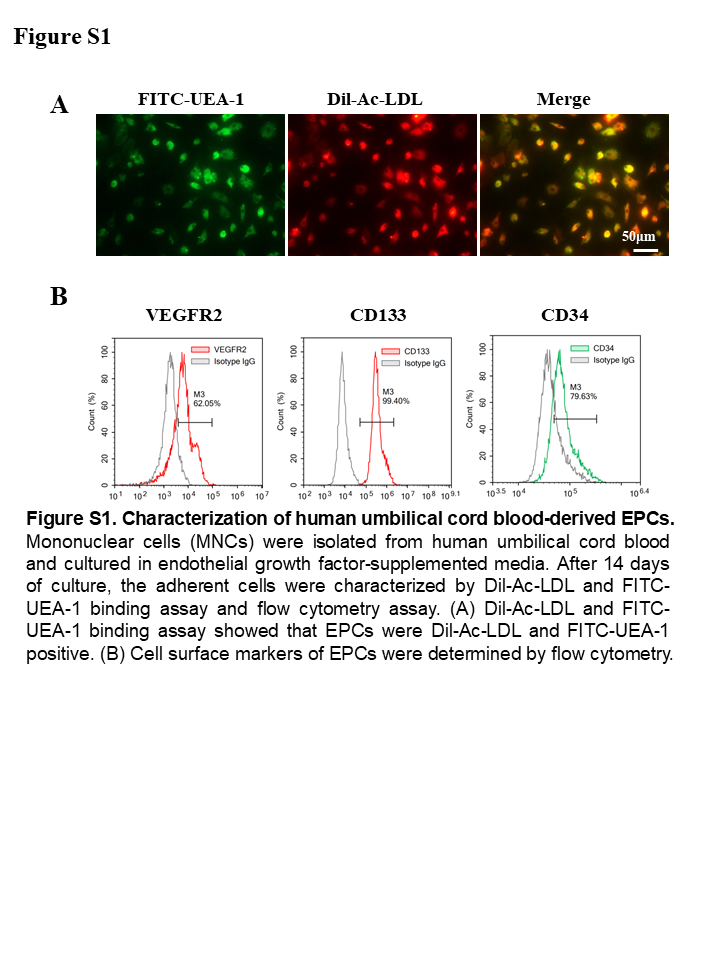

Supplement: Supplementary file 1 — Figure S1 [file JCMM-25-652-s001.tif]

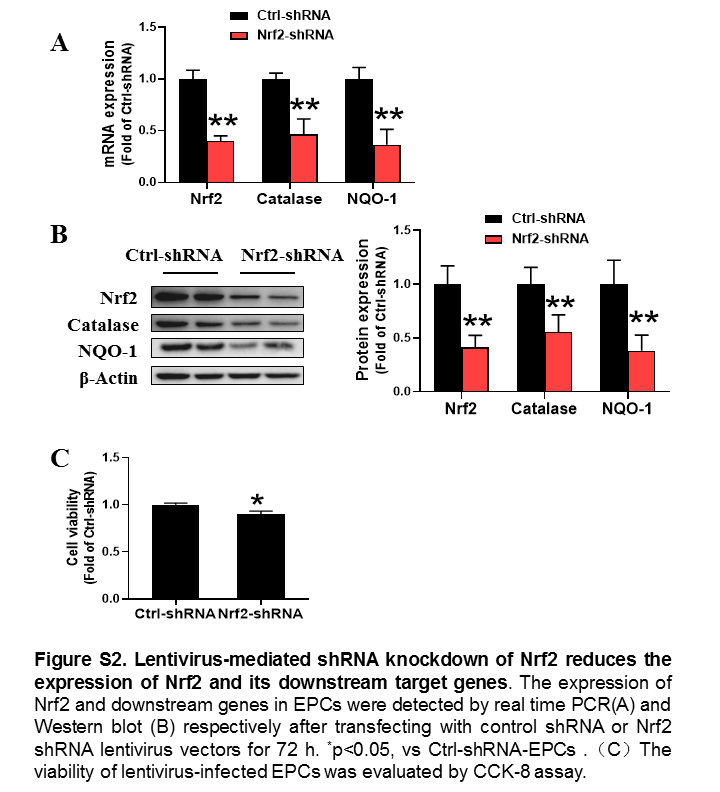

Supplement: Supplementary file 2 — Figure S2 [file JCMM-25-652-s002.TIF]

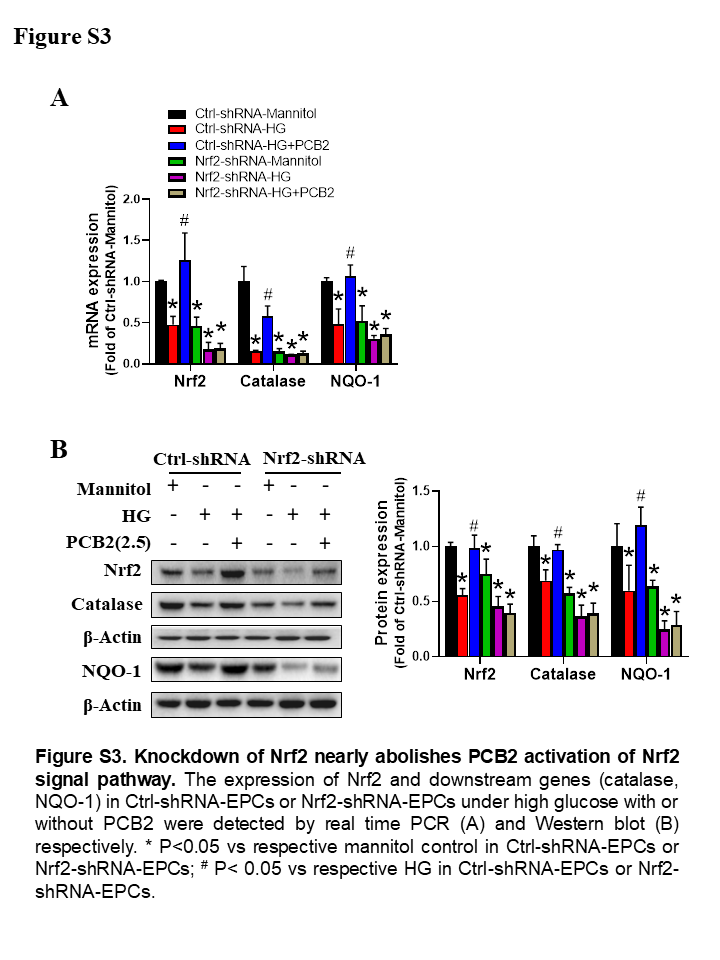

Supplement: Supplementary file 3 — Figure S3 [file JCMM-25-652-s003.tif]
